# Cross-Dysregulation of *O*-GlcNAcylation and PI3K/AKT/mTOR Axis in Human Chronic Diseases

**DOI:** 10.3389/fendo.2018.00602

**Published:** 2018-10-09

**Authors:** Ninon Very, Anne-Sophie Vercoutter-Edouart, Tony Lefebvre, Stéphan Hardivillé, Ikram El Yazidi-Belkoura

**Affiliations:** Université Lille, CNRS, UMR 8576—UGSF–Unité de Glycobiologie Structurale et Fonctionnelle, Lille, France

**Keywords:** *O*-GlcNAcylation, PI3K/AKT/mTOR, cancer, diabetes, cardiovascular, neurodegenerative diseases

## Abstract

The hexosamine biosynthetic pathway (HBP) and the phosphatidylinositol 3-kinase (PI3K)/AKT/mammalian target of rapamycin (mTOR) signaling pathway are considered as nutrient sensors that regulate several essential biological processes. The hexosamine biosynthetic pathway produces uridine diphosphate N-acetylglucosamine (UDP-GlcNAc), the substrate for *O*-GlcNAc transferase (OGT), the enzyme that *O*-GlcNAcylates proteins on serine (Ser) and threonine (Thr) residues. *O*-linked β-N-acetylglucosaminylation (*O*-GlcNAcylation) and phosphorylation are highly dynamic post-translational modifications occurring at the same or adjacent sites that regulate folding, stability, subcellular localization, partner interaction, or activity of target proteins. Here we review recent evidence of a cross-regulation of PI3K/AKT/mTOR signaling pathway and protein *O*-GlcNAcylation. Furthermore, we discuss their co-dysregulation in pathological conditions, e.g., cancer, type-2 diabetes (T2D), and cardiovascular, and neurodegenerative diseases.

## Introduction

**Figure 1:**
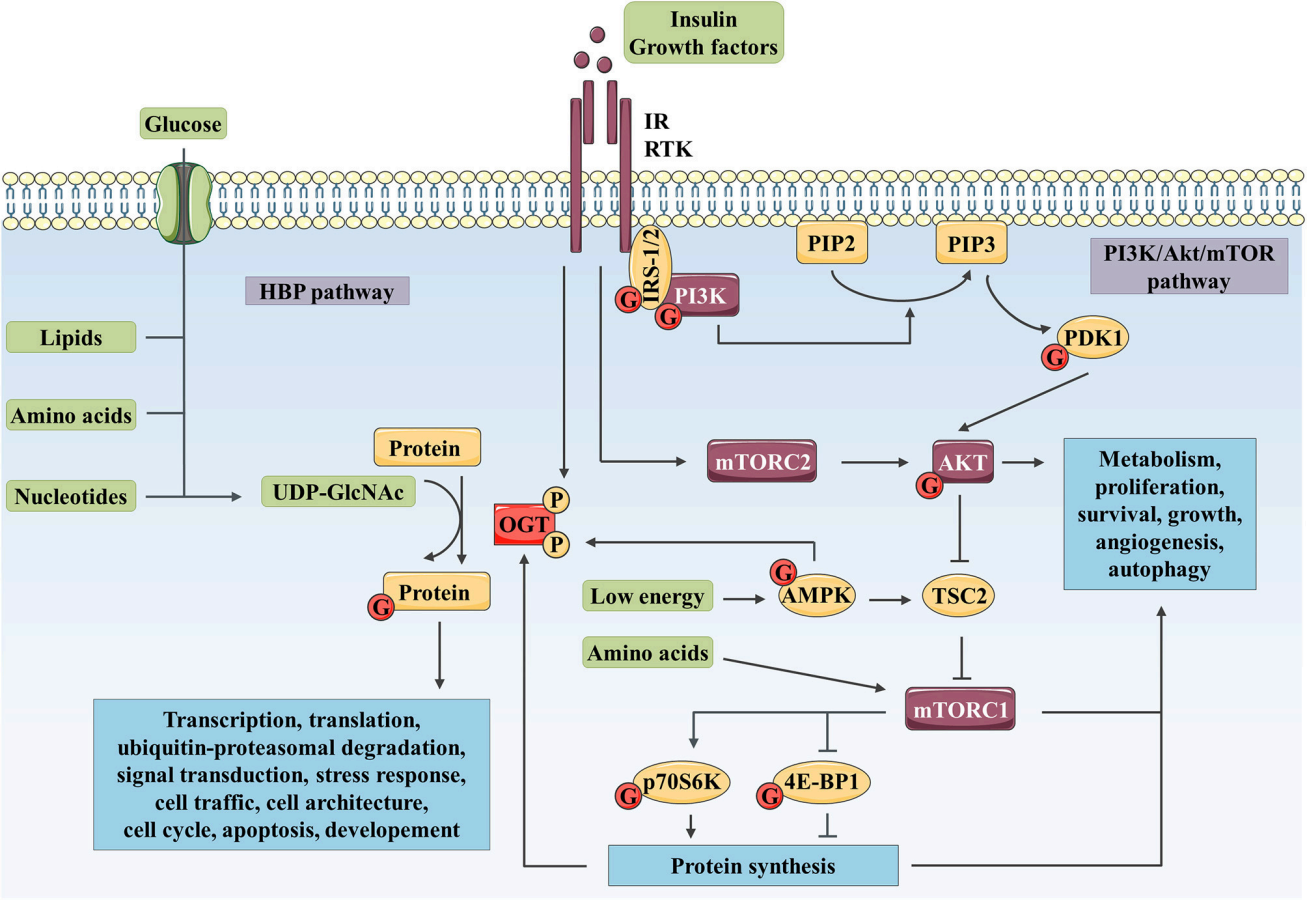
Complex interplay between *O*-GlcNAcylation and PI3K/AKT/mTOR signaling pathway controls numerous biological processes. The HBP integrates a fraction of the glucose entering the cell as well as lipid, nucleotide, and amino acid metabolites to produce UDP-GlcNAc. Then, OGT uses UDP-GlcNAc as a nucleotide sugar donor substrate to add a GlcNAc group on serine and threonine residues of target proteins. Like phosphorylation, *O*-GlcNAcylation is a dynamic and reversible post-translational modification. Its targets are involved in a wide range of biological processes such as transcription, translation, ubiquitin-proteasomal degradation, signal transduction, cell traffic and architecture, cell cycle, apoptosis or development. In parallel, binding of insulin or growth factor to their RTK leads to receptor activation and recruitment of IRS-1/2 and PI3K. PI3K produces PIP3 (from PIP2), which recruits AKT and PDK1 to the plasma membrane. PDK1 and mTOR in mTORC2 activate AKT through phosphorylation. mTORC1 is activated by AKT through TSC2 inhibition and upon amino acid stimulation and, is inhibited in response to low energy by AMPK. mTORC1 promotes protein synthesis via direct phosphorylation of p70S6K and 4E-BP1. By phosphorylating key substrates, AKT and mTORC1 regulate metabolism, cell cycle, proliferation, survival, growth, angiogenesis and autophagy. OGT localization and activity are regulated through phosphorylation by IR and AMPK. OGT stability is indirectly regulated at the protein synthesis level *via* mTORC1. Reciprocally, several actors of the PI3K/AKT/mTOR signaling pathway are modified by *O*-GlcNAcylation such as IRS-1, PI3K, PDK1, AKT, AMPK, p70S6K, and 4E-BP1.

*O*-linked β-N-acetylglucosaminylation (*O*-GlcNAcylation) is a dynamic modification of serine (Ser) and threonine (Thr) amino acids of cytoplasmic, nuclear (1), and mitochondrial (2) proteins with a single residue of N-acetylglucosamine (GlcNAc). This post-translational modification is controlled by two single antagonist enzymes: *O*-GlcNAc transferase (OGT) and *O*-GlcNAcase (OGA), which transfer and remove the GlcNAc moiety, respectively. The nucleotide sugar donor, uridine diphosphate N-acetylglucosamine (UDP-GlcNAc), is the final product of the hexosamine biosynthetic pathway that is at the nexus of glucose, amino acid, lipid, and nucleotide metabolisms (Figure 1). Consequently, *O*-GlcNAcylation is considered as a cellular nutrient sensor linking nutrient availability to intracellular signaling and biological responses. To date, thousands of *O*-GlcNAcylated proteins endowing a wide range of functions have been identified and most of them are also phosphoproteins (3). In fact, *O*-GlcNAcylation and phosphorylation can modulate each other at the same or adjacent sites (4).

Over the last decade, emerging evidence has indicated that a cross-talk exists between *O*-GlcNAcylation and phosphatidylinositol 3-kinase (PI3K)/AKT/mammalian target of rapamycin (mTOR) signaling pathway (5). The PI3K/AKT/mTOR signaling pathway is a key transducer of metabolic and mitogen signals (such as energy, amino acids, insulin or growth factors) that modulate gene expression, protein translation and cellular metabolism, thus regulating cell growth and proliferation (Figure 1). Aberrant activation of this signaling pathway as well as altered protein *O*-GlcNAcylation have both described in several types of cancer, cardiovascular and metabolic diseases, aging, and neurodegeneration in human (6–8). This mini-review summarizes and discusses recent evidence linking cross-regulation and co-dysregulation of *O*-GlcNAcylation and PI3K/AKT/mTOR signaling pathway in physiological conditions and in human chronic diseases, respectively.

## The PI3K/AKT/mTOR signaling pathway and its cross-regulation with protein *O*-GlcNAcylation

Binding of insulin or growth factors to their plasma membrane tyrosine kinase receptors (RTK) triggers the phosphorylation of PI3K, either directly by the RTK or indirectly *via* phosphorylation of adapter signaling proteins such as insulin receptor substrate-1 or 2 (IRS-1/2; Figure 1). Phospho-PI3K catalyzes the formation of membrane phosphatidylinositol-3,4,5-trisphosphate (PIP3) which drives the activation of the phosphoinositide-dependent protein kinase-1 (PDK-1) and the recruitment of AKT. The latter is partially activated through initial phosphorylation at Thr308 by PDK-1 and fully activated after phosphorylation at Ser473 by the mTOR complex 2 (mTORC2) (9). Once activated, AKT phosphorylates several downstream effectors [e.g., mTOR, forkhead box proteins O (FoxO), glycogen synthase kinase 3 β (GSK3β), BCL-2-associated agonist of cell death (BAD) or endothelial nitric oxide synthase (eNOS)] that in return regulate and coordinate a variety of cellular responses including cell proliferation, survival and growth, glucose metabolism, and angiogenesis (9). Tuberous sclerosis complex 2 (TSC2), inhibited by AKT-dependent phosphorylation, is a critical negative regulator of mTOR complex 1 (mTORC1). mTORC1 induces protein synthesis through phosphorylation of eukaryotic translation initiation factor 4E (eIF4E)-binding protein-1 (4E-BP1) and ribosomal protein S6 kinase (p70S6K) (9). mTORC1 also regulates nucleotide, lipid and glucose metabolisms, angiogenesis and autophagy processes by regulating alpha-activating transcription factor 4 (ATF4), lipin-1, hypoxia-inducible factor-1α (HIF-1α) or Unc-51 like autophagy activating kinase 1 (ULK1) (10, 11). In response to amino acid stimulation, mTORC1 is recruited to the lysosomal surface where it is activated by Ras homolog enriched in brain (Rheb) (9). Upon elevated AMP/ATP ratio, AMP-activated protein kinase (AMPK) phosphorylates, and activates TSC2 leading to inhibition of mTORC1 activity (10, 11).

In parallel, *O*-GlcNAcylation targets proteins involved in transcription, translation, ubiquitin-proteasomal degradation, signal transduction, stress response, cellular trafficking and architecture, cell cycle, apoptosis, and development (12). OGT activity is sensitive to UDP-GlcNAc levels, thus, addition of glucose or glucosamine globally increases levels of *O*-GlcNAcylation (13).

Many studies have established a complex interplay between PI3K/AKT/mTOR signaling pathway and protein *O*-GlcNAcylation (Figure 1). After insulin stimulation, the C-terminal PIP-binding domain of OGT (PPO) allows its translocation from the nucleus to the plasma membrane in murine 3T3-L1 adipocytes (14) and African green monkey COS-7 fibroblasts (15), and possibly to lipid rafts as observed in the human hepatic cancer cell line HepG2 (16). This translocation possibly facilitates the tyrosine phosphorylation of OGT by the insulin receptor (IR), which increases its enzymatic activity (17). The cellular energy sensor AMPK also regulates OGT. AMPK phosphorylates OGT at Thr444, which induces its nuclear translocation in differentiated C2C12 skeletal muscle myotubes (18) and promotes its dissociation from chromatin in human embryonic kidney 293T cells (19). In HepG2 cells, it has been further shown that OGT phosphorylation by AMPK inhibits histone H2B *O*-GlcNAcylation and gene transcription (19). In contrast, OGT targets several actors from the PI3K/AKT/mTOR signaling pathway, including IRS-1 (17, 20–24), PI3K (23), PDK1 (17), AKT (21, 25–27), AMPK (18, 19), 4E-BP1 (28), and p70S6K (29). Indeed, these proteins are *O*-GlcNAc-modified in IR and insulin growth factor-1 receptor (IGF-1R) expressing cell types including adipocytes, myocytes, hepatocytes, pancreatic beta (β) cells, endothelial cells, kidney and retina cells (30). However, only few studies have investigated the molecular impacts of *O*-GlcNAcylation on PI3K/AKT/mTOR signaling pathway and the subsequent biological effects under physiological conditions. *O*-GlcNAc modification of IRS-1 and AKT inhibits their activity either by disruption of their interaction with PI3K and PDK1 kinases, respectively, in 3T3-L1 adipocytes and MCF-7 breast cancer cell lines (17, 26), either by a “Yin-Yang” competition mechanism with activating phosphorylation as described in rat primary adipocytes and INS-1 pancreatic β cell lines (25, 27). *O*-GlcNAcylation also enhances 4E-BP1 stability *in vitro* in rat retinal TR-MUL Müller cells an *in vivo* in murine retinal cells, potentially by preventing its phosphorylation-dependent ubiquitin-mediated degradation (28). Protein *O*-GlcNAcylation could hence potentiate cellular nutrient sensing capacity of the PI3K/AKT/mTOR signaling pathway in order to regulate crucial intracellular processes.

## *O*-GlcNAcylation and PI3K/AKT/mTOR signaling pathway cross-dysregulation in human diseases

### Cancer

The Warburg effect is a metabolic reprogramming of the cell from oxidative phosphorylation to aerobic glycolysis that allows energy production and *de novo* macromolecule synthesis required to sustain cancer cells proliferation and growth. Enhanced glucose and glutamine uptake observed in the Warburg effect would lead to an increased flux through HBP and the hyper-*O*-GlcNAcylation that has been observed in many cancers (31). Aberrantly activated PI3K/AKT/mTOR signaling pathway is known to play a central role in aerobic glycolytic reprogramming, tumor growth, and survival (32), and a cross-talk between PI3K/AKT/mTOR signaling and *O*-GlcNAcylation has been observed in several cancers.

Insulin or serum growth factors stimulation lead to increased OGT expression in a PI3K-dependent manner in HepG2 and MCF-7 cell lines (16, 33). Although it was not investigated in these studies, it is likely that this effect could be related to mTOR activation. Since it was observed that pharmacological inhibition of mTOR enhances proteasomal and autophagic degradation of OGT in HepG2 cells (34). We have also demonstrated that inhibition of mTOR affects OGT protein level and overall *O*-GlcNAcylation levels in HCT116 colon cancer cell line (35). In breast cancer cell lines the positive regulation of OGT expression through mTOR is dependent on c-Myc-induced heat shock protein 90A (HSP90A) transcription (36). This chaperone binds to OGT and prevents its proteasomal degradation (36). Additionally, the transcriptional regulator Yes-associated protein (YAP) strongly activates the OGT promoter in hepatic cancer cell lines. In turn, *O*-GlcNAcylation of YAP promotes its stability, and its tumorigenic activity both *in vitro* and *in vivo* in liver cancer mouse models showing that a positive feedback is set up in liver tumorigenesis (37). YAP is activated by PI3K in hepatocellular (38) and mammary carcinoma (39), but has been shown to regulate PI3K/AKT/mTOR signaling in the MCF 10A human immortalized mammary epithelial cell line (40). These recent works highlight once more the tight link that exists in cancer cells between PI3K/AKT/mTOR axis and OGT activity.

**Figure 2:**
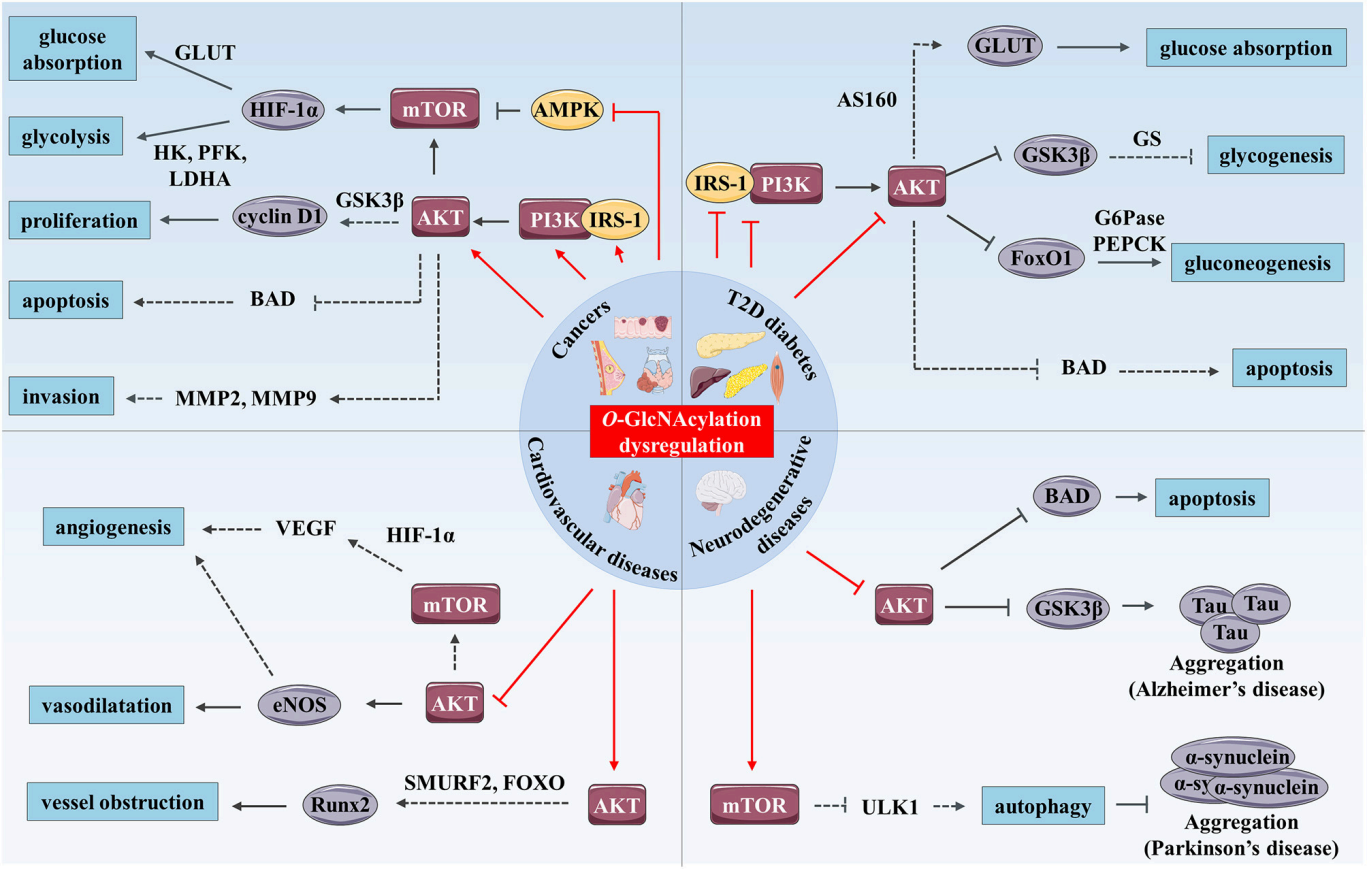
*O*-GlcNAcylation and PI3K/AKT/mTOR signaling pathway cross-dysregulation in human chronic diseases. *O*-GlcNAcylation dysregulation (represented by red arrows) modulates the PI3K/AKT/mTOR signaling pathway and promotes development of human chronic diseases such as cancer, T2D and cardiovascular and neurodegenerative diseases. In cancer, increased *O*-GlcNAcylation stimulates the PI3K/AKT/mTOR signaling pathway by up-regulating expression and activity of IRS-1, PI3K, and AKT and by inhibiting AMPK. mTOR enhances glucose absorption and glycolysis through stimulation of expression of the transcription factor *HIF-1*α and its target genes such as *GLUTs, HK, PFK*, and *LDHA*. AKT enhances cancer cell proliferation through increased cyclin D1 expression possibly through GSK3β inhibition. Moreover, AKT has anti-apoptotic and pro-invasion activities and these effects may result in the regulation of several targets including BAD, MMP-2, and MMP-9. In T2D, increased *O*-GlcNAcylation associated with hyperglycemia promotes insulin resistance in skeletal muscle, liver and adipose tissue by inhibition of IRS-1/PI3K interaction and down-regulation of IRS-1 and AKT activity. AKT inhibition induces reduced glucose absorption through down-regulation of GLUT4 translocation to the plasma membrane which might be mediated by AS160. Moreover, repression of AKT inhibits glycogenesis and stimulates gluconeogenesis through regulation of GSK3β, FoxO1, and respective targets (GS, G6Pase, and PEPCK). Additionally, *O*-GlcNAc-mediated AKT inhibition induces pancreatic β cell apoptosis. In hyperglycemic or diabetic cardiovascular tissues, *O*-GlcNAcylation reduces vasodilatation vessels and angiogenesis via inhibition of AKT, and probably eNOS and VEGF. Enhanced *O*-GlcNAcylation in these tissues could also promote calcification and therefore vessel obstruction through *Runx2* overexpression possibly mediated by AKT regulation of SMURF2 and/or FoxO. Finally, increased *O*-GlcNAcylation stimulates Tau and α-synuclein aggregations involved in AD and Parkinson's disease respectively. These processes are mediated by AKT inhibition and Tau kinase GSK3β activation in AD, and by mTOR activation and reduced autophagy in Parkinson's disease, which could result from ULK1 inhibition. Furthermore, *O*-GlcNAc-mediated AKT repression promotes neural cell apoptosis in part, by up-regulation of BAD.

*O*-GlcNAcylation impacts PI3K/AKT and mTOR axis in cancer cells. Pre-B acute lymphocytic leukemia (pre-B-ALL) cells overexpress OGT and exhibit a higher *O*-GlcNAcylation levels and an overactivation of PI3K, AKT and c-Myc compared to normal B cells. This dysregulation is associated with the overexpression of the transcription factor *HIF-1*α and its target glycolytic genes such as *glucose transporter 1* (*GLUT1*), *hexokinase 2* (*HK2*), *phosphofructokinase-1* (*PFK-1*) and *lactate dehydrogenase A* (*LDHA*). *OGT* knockdown, in pre-B-ALL cells, decreases PI3K and AKT activation and glycolysis, resulting in a reduced cell proliferation and apoptosis. These inhibitory effects can be partly rescued by IGF-1 mediated stimulation of PI3K/AKT, indicating that effect of OGT on glycolysis is, in part, PI3K/AKT-dependent (41). Similarly, in 3D cultures of T4-2 breast cancer cells, OGT inhibition or silencing suppresses AKT signaling and glycolytic activity (42) (Figure 2).

In addition to glycolysis, regulation of PI3K/AKT signaling by *O*-GlcNAcylation was shown to modulate proliferation, growth and invasion properties of cancer cells (32, 42–47). We have demonstrated that knockdown or pharmacological inhibition of OGT decreases PI3K activation and prevents serum-stimulated cyclin D1 synthesis, leading to a delay in proliferation of MCF-7 cells (33). Since AKT prevents ubiquitin-mediated degradation of cyclin D1 by inhibiting GSK3β activity in the murine NIH/3T3 fibroblast cell line (43), it is likely that the decrease in cyclin D1 level could result from an increase of its proteasomal degradation under low *O*-GlcNAcylation levels. Reciprocally, enhanced *O*-GlcNAcylation level stimulates PIP3 production and AKT phosphorylation in MCF-7 cells (44). Similar results showed that hyper-*O*-GlcNAcylation induced by *OGA* down-regulation in 8305C thyroid anaplastic tumor cell line stimulates proliferation through increased phosphorylation of AKT at Ser473 and cyclin D1 amount (45). Additionally, glucose deprivation in osteosarcoma U2OS cell line attenuates protein *O*-GlcNAcylation, phosphorylation of IRS-1 and AKT, production of PIP3 and suppresses cell growth (46). Importantly, in these cell line, insulin signaling pathway, and tumor growth can be rescued by glucosamine-mediated increased HBP flux and *O*-GlcNAcylation (46). In parallel, increased *O*-GlcNAcylation promotes gastric and thyroid cancer cells invasion in a PI3K/AKT dependent manner, since the pro-invasion effect of *O*-GlcNAcylation is suppressed by PI3K inhibition or *AKT* silencing (47, 48). This may result from the regulation that *O*-GlcNAcylation exerts on AKT-mediated control of a myriad of downstream substrates, such as matrix metalloproteinase-2 (MMP-2) and MMP-9 (49) (Figure 2). However, other studies report contradictory results regarding the effect of *O*-GlcNAcylation on the activation of AKT signaling pathway (26, 50). *OGA* overexpression reduces AKT *O*-GlcNAcylation and promotes its activation, albeit in a PI3K-independent manner, both in HepG2 cells and in liver of euglycemic mice (50). Our group also demonstrated that *OGT* silencing prevents AKT Ser473 phosphorylation in HepG2 (16) and MCF-7 (33) cell lines. More recently, this effect has also been described in cholangiocarcinoma cell lines (51). Furthermore, AKT *O*-GlcNAcylation at Thr305 and Thr312 reduces MCF-7 cell proliferation and migration *via* inhibition of AKT phosphorylation at Thr308 and disruption of its interaction with PDK1 (26).

Finally, it was shown that *O*-GlcNAcylation regulates the mitogenic mTOR signaling pathway through targeting the mTOR inhibitor AMPK (35, 52, 53). Increased *O*-GlcNAcylation in colon cancer cells, either by *OGT* overexpression or OGA inhibition, reduces phosphorylation of AMPK at Thr172, activates mTOR and induces cell growth *in vitro* in LoVo cell line and *in vivo* in LoVo cell-derived tumors of BALB/c-nu/nu mice (52). We have confirmed that *O*-GlcNAcylation activates mTOR in HCT116 colon cancer cell line but not in CCD841CoN normal cells (35). Reciprocally, *OGT* silencing or inhibition increases phosphorylation of AMPK, decreases phosphorylation of mTOR downstream effectors 4E-BP1 and p70S6K, decreases *HIF-1a, GLUT1*, and *LDHA* expression and impairs glucose uptake and growth in breast cancer cell lines (53) (Figure 2).

Together, these studies establish the involvement of *O*-GlcNAcylation in cancer biology (increased glycolysis, proliferation, growth, and invasion) through direct activation of the PI3K/AKT/mTOR axis. One may consider this post-translational modification as a key node between metabolism and cell signaling. However, intricate ties linking metabolism and cancer are not completely elucidated and need further investigations. In parallel, anti-cancer inhibitors targeting mTOR axis are currently in clinical development and must be encouraged (54). Tumor cells resistant to GDC-0941, a PI3K inhibitor, exhibit an increased activation of the PI3K/AKT/mTOR signaling pathway and *OGT* expression in comparison to GDC-0941-sensitive cells. Interestingly, *OGT* silencing sensitizes these cells to GDC-0941 (55). In this sense, targeting OGT in cancer cells and/or adapting patients to low caloric diet could increase the efficiency of anti-PI3K/AKT/mTOR therapeutic strategies and foil drug resistance.

### Type 2 diabetes

Insulin resistance, a hallmark of type 2 diabetes (T2D), refers to impaired insulin sensitivity and glucose uptake of target tissues (liver, skeletal muscle, and adipose tissue). PI3K/AKT signaling pathway plays a key role in the regulation of glucose homeostasis by inhibiting gluconeogenesis and activating glycogenesis *via* the inhibition of FoxO1 and GSK3β respectively (9) (Figure 2). Some studies have also established a link between dysregulation of *O*-GlcNAcylation cycling and insulin resistance.

Interestingly, single nucleotide polymorphisms (SNPs) on *OGA* (also called *MGEA5* for *meningioma expressed antigen 5*) gene and *GFPT2* (for *GFAT isomerizing 2*) gene, coding the glutamine fructose-6-phosphate amidotransferase (GFAT) rate-limiting enzyme controlling the production of UDP-GlcNAc, are associated with increased T2D risk in American-Mexican and Caucasian populations, respectively (56, 57). These mutations may lead to reduced *OGA* expression and increased *GFPT2* expression respectively (56, 57), and an up-regulation of cellular *O*-GlcNAcylation levels. *O*-GlcNAcylation levels are significantly increased in skeletal muscle, liver, heart, colon-rectum, erythrocytes, and leukocytes of diabetic animals and humans (35, 58–62). Consistent with these epidemiologic data, *db/db* mice overexpressing *Oga* showed improved hepatic insulin sensitivity (63), whereas *Ogt* overexpression and subsequent elevation of global *O*-GlcNAcylation level inhibits insulin signaling pathway, both *in vitro* in 3T3-L1 adipocyte and Fao hepatic cell lines, and *in vivo* in skeletal muscle and adipose tissue in mice (15, 64, 65). Skeletal muscle-specific *Ogt* knockout mice have increased glucose uptake, insulin signaling and whole-body insulin sensitivity (62). Likewise, inhibition of OGA with *O*-(2-acetamido-2-deoxy-D-glucopyranosylidene) amino-N-phenylcarbamate (PUGNAc) induces insulin resistance in 3T3-L1 and rat primary adipocytes by perturbing both insulin-signaling pathway and glucose absorption (17, 21). Indeed, blockade of OGA increases *O*-GlcNAcylation of IRS-1 and AKT while decreasing their phosphorylation (17, 21). Consequently, a reduction of insulin-stimulated PI3K/IRS-1 interaction, GSK3β phosphorylation (15, 17, 64) and GLUT4 translocation to the plasma membrane is observed (17, 21). This reduced translocation of GLUT4 might be related to the decrease in AKT phosphorylation since phosphorylation of AKT substrate of 160 kDa (AS160) is required for insulin-stimulated translocation of GLUT4 to the plasma membrane (66). Reciprocally, in euglycemic HepG2 and mice hepatic cells, the reduced global *O*-GlcNAcylation levels induced by *OGA* overexpression is associated with an increase of AKT activation but not of PI3K (50). This results in inhibition by phosphorylation of GSK3β (Ser9) and FoxO1 (Ser166), leading to a decrease of gluconeogenic genes transcription, including *glucose-6-phosphatas*e (*G6Pase*) and *phosphoenolpyruvate carboxykinase* (*PEPCK*) (50). In addition, glycogen synthase (GS), substrate of GSK3β, is also *O*-GlcNAc-modified in 3T3-L1 cells and this modification blocks its activation, which is associated with insulin resistance (67). These data clearly establish the impact of *O*-GlcNAcylation in the etiology of insulin resistance and, thus, potentially in metabolic related diseases such as diabetes.

However, there are studies showing that OGA inhibition does not cause insulin resistance in 3T3-L1 adipocytes, rat liver and muscle (68, 69), while others show that OGA inhibition induces insulin resistance in rat skeletal muscle in an AKT and GSK3β-independent manner (70). In these studies, authors suggest that conflicting primary results might result from the use of the non-selective OGA inhibitor PUGNAc which has been shown to also inhibit lysosomal hexosaminidases and alter plasma membrane oligosaccharide structures that are critical in signal transduction (71, 72). These contradictory findings could also be due to supraphysiological concentrations of insulin (12 nmol/L) used for stimulation (70). These findings raise the question of whether high *O*-GlcNAcylation levels are responsible for insulin resistance and show to what extent the understanding of the role of *O*-GlcNAcylation in cell signaling regulation in such multifactorial disease needs to be deepened.

In addition to its role in insulin resistance, *O*-GlcNAcylation could also take part in pancreatic islet β cell dysfunction. Pancreatic β cells are the cells in the body in charge of producing, storing and releasing insulin upon increased blood glucose concentration; its dysregulation is a cause of diabetes. OGT and global *O*-GlcNAcylation levels are increased in pancreatic islets of Goto-Kakizaki diabetic rats (73). In murine pancreatic β cells, glucosamine-mediated hyperglycemia increases *O-*GlcNAcylation of AKT and concomitantly reduces its Ser473 phosphorylation (27). Glucosamine induces β cells apoptosis likely through *O*-GlcNAc-mediated inhibition of AKT (27) (Figure 2). In contrast, β cell-specific *Ogt* knockout mice develop β cell failure and diabetes. In this model, a reduction of AKT phosphorylation at Ser473 was observed (74). These data suggest that the phospho/*O*-GlcNAc interplay on AKT may play a pivotal role as a regulator of downstream signaling cascades in response to nutrient conditions. The impact of *O*-GlcNAcylation dysregulation may be tissue-specific (75). In conclusion, increased *O*-GlcNAcylation in diabetes toward PI3K/AKT-mediated insulin resistance in target tissues could contribute to the maintenance of the pathology.

### Cardiovascular diseases

Many studies suggest that elevated protein *O*-GlcNAcylation levels contribute to cardiovascular complications (76). Chronic hyperglycemia is a risk factor for cardiovascular diseases and patients with diabetes may develop atherosclerotic carotid plaques with a marked increase of *O*-GlcNAcylation levels (23). Aorta from streptozotocin-induced hyperglycemic mice exhibits high levels of *O*-GlcNAcylation and impaired vascular sprouting (77). Endothelial dysfunction is a feature of cardiovascular diseases that is characterized by reduced bioavailability of nitric oxide (NO) produced by endothelial nitric oxide synthase (eNOS). Endothelial production of NO plays indeed a key role in preventing vascular diseases by preventing thrombosis, inflammation, vascular tone, and remodeling (78). *O*-GlcNAc modification is known to modulate NO production in endothelial cells, promoting macro- and microvascular complications. In response to insulin, AKT induces vasodilatation in primary human aortic endothelial cells (HAEC), and it may exert anti-atherogenic effects by increasing activating phosphorylation of eNOS at Ser615 and Ser1177 (79) (Figure 2). Federici and collaborators showed that hyperglycemia or HBP activation decreases eNOS activity through a reduction of AKT and eNOS phosphorylation in human coronary artery endothelial cells (HCAEC) (23). *In vitro*, glucosamine-induced protein *O*-GlcNAcylation also modulates the angiogenic properties of EA.hy926 endothelial cells, most probably by a concomitant increase of AKT *O*-GlcNAcylation that leads to inhibition of its pro-angiogenic activity (77). AKT could directly up-regulate the production of the pro-angiogenic factor NO (80). In addition, PI3K/AKT/mTOR signaling pathway stimulates angiogenesis by increasing expression of *HIF-1*α and its target, the *vascular endothelial growth factor* (*VEGF*) (80) (Figure 2). Elevated *O*-GlcNAcylation levels also induce vascular calcification *in vitro* in murine cells, and *in vivo* in aortic arc and descending aorta of diabetic mice. It has been shown, in primary mouse vascular smooth muscle cells (VSMC), that this process results from increased Thr430/Thr479-AKT *O*-GlcNAcylation, which promotes its activation and the expression of *osteogenic runt-related transcription factor 2* (*Runx2*) (81). AKT-mediated Runx2 stabilization by degradation of E3 ubiquitin ligase SMURF2 or by the nuclear exclusion of its transcription regulators FoxO could take part in this mechanism (82) (Figure 2). Thus, angiogenesis impairment and vessel obstruction are among the biological effects related to aberrant O-GlcNAcylation of AKT-mediated signaling involved in cardiovascular complications associated with diabetes.

### Neurodegenerative diseases

Dysregulated *O*-GlcNAcylation has been implicated in the pathogenesis of neurodegenerative disorders such as Alzheimer's disease (AD) and Parkinson's disease (83). Neurofibrillary degeneration associated with aggregation of abnormal hyperphosphorylated tubulin-associated unit (Tau) proteins is one the features of AD. The latter undergoes a “Ying-Yang” competition mechanism between *O*-GlcNAcylation and phosphorylation (83). Using thiamet-G, a blood-brain barrier-permeable OGA inhibitor, several *in vivo* studies evidenced the ability of *O*-GlcNAcylation to protect against Tau aggregation (84–86). Increased levels of *O*-GlcNAcylation in mice brain by intracerebroventricular injection of thiamet-G is associated with Tau site-dependent increased and decreased phosphorylation further confirming the complex relation between modifications on Tau protein (87). Elevated phosphorylation of Tau at Ser199, Ser202, Ser396, and Ser422 is likely to result from the combination of increased Tau *O*-GlcNAcylation, PI3K-independent inhibition of Ser473-AKT phosphorylation and the subsequent over-activation of GSK3β, a key Tau kinase (87) (Figure 2). Elevated *O*-GlcNAcylation of proteins is found in Parkinson's disease postmortem brains (88). In rat primary cortical neurons, thiamet-G treatment increases accumulation of α-synuclein, a neuronal protein that aggregates in this pathology, through activation of mTOR and reduction of autophagy (88) (Figure 2). Conversely, α-synuclein is *O*-GlcNAcylated at Thr72 and Ser87, leading to reduced aggregation *in vitro* (89, 90). But, these discrepancies could be due to different experimental approaches. Evidence that excessive *O*-GlcNAcylation is detrimental to neurons by increasing α-synuclein accumulation was demonstrated *in vitro* and related to mTOR pathway (88), while *O*-GlcNAc-reduced aggregation of α-synuclein was demonstrated by biochemical approaches (89, 90). Taken together, these results indicate that the mitigation of pathological aggregation of neuronal proteins by direct *O*-GlcNAc modification is a complex mechanism that could be indirectly counterbalanced by AKT/mTOR signaling pathway.

Another common pathological hallmark of neurodegenerative diseases is the loss of neurons as a consequence of neuronal cell death (91). Although not yet studied in such pathological conditions, indirect evidence suggests that *O*-GlcNAcylation could be involved in the regulation of neuronal apoptosis. Elevation of protein *O*-GlcNAcylation after cerebral ischemia is responsible for *O*-GlcNAc-mediated AKT inhibition, BAD activation and neuronal apoptosis in mice (25). An increase of *O*-GlcNAcylation levels is also associated with a default in Thr308-AKT phosphorylation and cellular apoptosis during cortical differentiation of human embryonic stem cells (hESC) (92) (Figure 2). These studies strongly support that *O*-GlcNAc-mediated AKT inhibition might be involved in neuronal cell loss of function and apoptosis in neurodegenerative diseases.

## Conclusion

Highlighted by the studies discussed above *O*-GlcNAcylation and the PI3K/AKT/mTOR signaling pathway appear to be intimately cross-linked. Both are considered as metabolic sensor that regulate folding, stability, subcellular localization, partner interaction, and therefore the activity of a plethora of targets involved in key biological functions. Here, we summarized evidence that *O*-GlcNAcylation can modulate the activation of the PI3K/AKT/mTOR signaling pathway by targeting different signaling actors, and that, reciprocally; expression, localization and activation of OGT are regulated by these signaling pathways (Figure 1). Although, further works are required to clarify the roles of *O*-GlcNAcylation on PI3K/AKT/mTOR regulation under normal physiological context, their interplay is highlighted by their associated dysregulation in several types of cancer, T2D, and cardiovascular and neurodegenerative diseases (Figure 2). Under pathological glucose conditions, aberrant *O*-GlcNAcylation levels result in activation or inhibition PI3K/AKT/mTOR signaling pathway as found in cancer and diabetes, respectively. Because of the key role of the PI3K/AKT/mTOR signaling pathway in cellular metabolism and physiology, these regulatory mechanisms contribute to pathogenicity by promoting, on one hand, glycolysis, proliferation, growth and invasion of cancer cells, and on the other hand, insulin resistance in insulin target tissues and/or pancreatic β cell dysfunction and death. Moreover, *O*-GlcNAc-mediated disturbance of AKT activity in endothelial cells leads to impairment of angiogenesis and vessel obstruction, supporting cardiovascular diseases associated with T2D. Finally, *O*-GlcNAcylation regulation of the PI3K/AKT/mTOR signaling pathway can indirectly modulate aggregation of neuronal proteins, such as Tau and α-synuclein that are involved in AD and Parkinson's disease, respectively, as well as in neuronal cell death. Taken together, evidence presented here shows that targeting OGT or OGA with selective small molecules to inhibit their activity or their interaction with specific actors of the PI3K/AKT/mTOR signaling pathway, in association with an adapted diet, may be a promising combined therapeutic approach to treat chronic metabolic-related diseases.

## Author contributions

NV and IE concepted the plan and wrote the review. A-SV-E, TL, and SH revised it critically for important intellectual content.

### Conflict of interest statement

The authors declare that the research was conducted in the absence of any commercial or financial relationships that could be construed as a potential conflict of interest.

## Abbreviations

4E-BP1: eukaryotic translation initiation factor 4E-binding protein-1
AD: Alzheimer's disease
AMPK: adenosine monophosphate-activated protein kinase
BAD: BCL-2-associated agonist of cell death
eNOS: endothelial nitric oxide synthase
FoxO: forkhead box protein O
GlcNAc: N-acetylglucosamine
GLUT: glucose transporter
GSK3β: glycogen synthase kinase 3 β
HBP: hexosamine biosynthetic pathway
HIF-1α: hypoxia-inducible factor-1α
IR: insulin receptor
IRS-1: insulin receptor substrate-1
mTOR: mammalian target of rapamycin
mTORC: mTOR complex
O-GlcNAcylation: O-linked β-N-acetylglucosaminylation
OGA: O-GlcNAcase
OGT: O-GlcNAc transferase
p70S6K: ribosomal protein S6 kinase
PDK-1: phosphoinositide-dependent protein kinase-1
PI3K: phosphatidylinositol 3-kinase
PIP3, phosphatidylinositol-3, 4: 5-trisphosphate
T2D: type-2 diabetes
Tau: tubulin-associated unit
UDP-GlcNAc: uridine diphosphate N-acetylglucosamine.

## References

[1] Kreppel LK Blomberg MA Hart GW. Dynamic glycosylation of nuclear and cytosolic proteins. Cloning and characterization of a unique O-GlcNAc transferase with multiple tetratricopeptide repeats. J Biol Chem. (1997) 272:9308–15. 9083067 10.1074/jbc.272.14.9308

[2] Hu Y Suarez J Fricovsky E Wang H Scott BT Trauger SA. Increased enzymatic O-GlcNAcylation of mitochondrial proteins impairs mitochondrial function in cardiac myocytes exposed to high glucose. J Biol Chem. (2009) 284:547–55. 10.1074/jbc.M808518200 19004814 PMC2610513

[3] Mishra S Ande SR Salter NW. O-GlcNAc modification: why so intimately associated with phosphorylation? Cell Commun Signal (2011) 9:1. 10.1186/1478-811X-9-1 21223562 PMC3023788

[4] Wang Z Gucek M Hart GW Cross-talk between O-GlcNAcylation and phosphorylation: site-specific phosphorylation dynamics in response to globally elevated O-GlcNAc. Proc Natl Acad Sci USA. (2008) 105:13793–8. 10.1073/pnas.0806216105 18779572 PMC2544533

[5] Józwiak P Forma E Bryś M Krześlak A. O-GlcNAcylation and Metabolic reprograming in Cancer. Front Endocrinol. (2014) 5:145. 10.3389/fendo.2014.00145 25250015 PMC4158873

[6] Das A Reis F Maejima Y Cai Z Ren J. mTOR signaling in cardiometabolic disease, cancer, and aging. Oxid Med Cell Longev. (2017) 2017:6018675. 10.1155/2017/6018675 28770023 PMC5523348

[7] Saxton RA Sabatini DM. mTOR signaling in growth, metabolism, and disease. Cell (2017) 168:960–76. 10.1016/j.cell.2017.02.004 28283069 PMC5394987

[8] Fruman DA Chiu H Hopkins BD Bagrodia S Cantley LC Abraham RT. The PI3K pathway in human disease. Cell (2017) 170:605–35. 10.1016/j.cell.2017.07.029 28802037 PMC5726441

[9] Manning BD Toker A. AKT/PKB signaling: navigating the network. Cell (2017) 169:381–405. 10.1016/j.cell.2017.04.001 28431241 PMC5546324

[10] Dibble CC Manning BD. Signal integration by mTORC1 coordinates nutrient input with biosynthetic output. Nat Cell Biol. (2013) 15:555–64. 10.1038/ncb2763 23728461 PMC3743096

[11] Antikainen H Driscoll M Haspel G Dobrowolski R. TOR-mediated regulation of metabolism in aging. Aging Cell (2017) 16:1219–33. 10.1111/acel.12689 28971552 PMC5676073

[12] Hart GW Slawson C Ramirez-Correa G Lagerlof O. Cross talk between O-GlcNAcylation and phosphorylation: roles in signaling, transcription, and chronic disease. Annu Rev Biochem. (2011) 80:825–58. 10.1146/annurev-biochem-060608-102511 21391816 PMC3294376

[13] Haltiwanger RS Blomberg MA Hart GW. Glycosylation of nuclear and cytoplasmic proteins. Purification and characterization of a uridine diphospho-N-acetylglucosamine:polypeptide beta-N-acetylglucosaminyltransferase. J Biol Chem. (1992) 267:9005–13. 1533623

[14] Whelan SA Lane MD Hart GW. Regulation of the O-Linked β-N-Acetylglucosamine transferase by insulin signaling. J Biol Chem. (2008) 283:21411–7. 10.1074/jbc.M800677200 18519567 PMC2490780

[15] Yang X Ongusaha PP Miles PD Havstad JC Zhang F So WV. Phosphoinositide signalling links O-GlcNAc transferase to insulin resistance. Nature (2008) 451:964–9. 10.1038/nature06668 18288188

[16] Perez-Cervera Y Dehennaut V Gil MA Guedri K Mata CJS Stichelen SO-V. Insulin signaling controls the expression of O-GlcNAc transferase and its interaction with lipid microdomains. FASEB J. (2013) 27:3478–86. 10.1096/fj.12-217984 23689613

[17] Whelan SA Dias WB Thiruneelakantapillai L Lane MD Hart GW. Regulation of insulin receptor substrate 1 (IRS-1)/AKT kinase-mediated insulin signaling by O-Linked β-N-acetylglucosamine in 3T3-L1 adipocytes. J Biol Chem. (2010) 285:5204–11. 10.1074/jbc.M109.077818 20018868 PMC2820748

[18] Bullen JW Balsbaugh JL Chanda D Shabanowitz J Hunt DF Neumann D. Cross-talk between two essential nutrient-sensitive enzymes. J Biol Chem. (2014) 289:10592–606. 10.1074/jbc.M113.523068 24563466 PMC4036179

[19] Xu Q Yang C Du Y Chen Y Liu H Deng M. AMPK regulates histone H2B O-GlcNAcylation. Nucleic Acids Res. (2014) 42:5594–604. 10.1093/nar/gku236 24692660 PMC4027166

[20] Patti ME Virkamäki A Landaker EJ Kahn CR Yki-Järvinen H. Activation of the hexosamine pathway by glucosamine *in vivo* induces insulin resistance of early postreceptor insulin signaling events in skeletal muscle. Diabetes (1999) 48:1562–71. 10426374 10.2337/diabetes.48.8.1562

[21] Park SY Ryu J Lee W. O-GlcNAc modification on IRS-1 and Akt2 by PUGNAc inhibits their phosphorylation and induces insulin resistance in rat primary adipocytes. Exp Mol Med. (2005) 37:220–9. 10.1038/emm.2005.30 16000877

[22] Klein AL Berkaw MN Buse MG Ball LE. O-linked N-acetylglucosamine modification of insulin receptor substrate-1 occurs in close proximity to multiple SH2 domain binding motifs. Mol Cell Proteomics (2009) 8:2733–45. 10.1074/mcp.M900207-MCP200 19671924 PMC2816021

[23] Federici M Menghini R Mauriello A Hribal ML Ferrelli F Lauro D. Insulin-dependent activation of endothelial nitric oxide synthase is impaired by O-linked glycosylation modification of signaling proteins in human coronary endothelial cells. Circulation (2002) 106:466–72. 10.1161/01.CIR.0000023043.02648.51 12135947

[24] Ball LE Berkaw MN Buse MG. Identification of the major site of O-linked β-N-acetylglucosamine modification in the C terminus of insulin receptor substrate-1. Mol Cell Proteomics (2006) 5:313–23. 10.1074/mcp.M500314-MCP200 16244361 PMC2435407

[25] Shi J Gu J Dai C Gu J Jin X Sun J. O-GlcNAcylation regulates ischemia-induced neuronal apoptosis through AKT signaling. Sci Rep. (2015) 5:14500. 10.1038/srep14500 26412745 PMC4585968

[26] Wang S Huang X Sun D Xin X Pan Q Peng S. Extensive Crosstalk between O-GlcNAcylation and Phosphorylation Regulates Akt Signaling. PLoS ONE (2012) 7:e0037427 10.1371/journal.pone.0037427 22629392 PMC3358304

[27] Kang E-S Han D Park J Kwak TK Oh M-A Lee S-A. O-GlcNAc modulation at Akt1 Ser473 correlates with apoptosis of murine pancreatic beta cells. Exp Cell Res. (2008) 314:2238–48. 10.1016/j.yexcr.2008.04.014 18570920

[28] Miller WP Mihailescu ML Yang C Barber AJ Kimball SR Jefferson LS. The translational repressor 4E-BP1 contributes to diabetes-induced visual dysfunction. Invest Ophthalmol Vis Sci. (2016) 57:1327–37. 10.1167/iovs.15-18719 26998719 PMC4811182

[29] Zeidan Q Wang Z De Maio A Hart GW. O-GlcNAc cycling enzymes associate with the translational machinery and modify core ribosomal proteins. Mol Biol Cell (2010) 21:1922–36. 10.1091/mbc.E09-11-0941 20410138 PMC2883937

[30] Wilcox G. Insulin and insulin resistance. Clin Biochem Rev. (2005) 26:19–39. 16278749 PMC1204764

[31] Ferrer CM Sodi VL Reginato MJ. O-GlcNAcylation in cancer biology: linking metabolism and signaling. J Mol Biol. (2016) 428:3282–94. 10.1016/j.jmb.2016.05.028 27343361 PMC4983259

[32] Courtnay R Ngo DC Malik N Ververis K Tortorella SM Karagiannis TC. Cancer metabolism and the Warburg effect: the role of HIF-1 and PI3K. Mol Biol Rep. (2015) 42:841–51. 10.1007/s11033-015-3858-x 25689954

[33] Olivier-Van Stichelen S Drougat L Dehennaut V El Yazidi-Belkoura I Guinez C Mir A-M. Serum-stimulated cell cycle entry promotes ncOGT synthesis required for cyclin D expression. Oncogenesis (2012) 1:e36. 10.1038/oncsis.2012.36 23552487 PMC3545199

[34] Park S Pak J Jang I Cho J. Inhibition of mTOR affects protein stability of OGT. Biochem Biophys Res Commun. (2014) 453:208–12. 10.1016/j.bbrc.2014.05.047 24858682

[35] Very N Steenackers A Dubuquoy C Vermuse J Dubuquoy L Lefebvre T. Cross regulation between mTOR signaling and O-GlcNAcylation. J Bioenerg Biomembr. (2018) 50:213–22. 10.1007/s10863-018-9747-y 29524020

[36] Sodi VL Khaku S Krutilina R Schwab LP Vocadlo DJ Seagroves TN mTOR/MYC Axis Regulates O-GlcNAc Transferase (OGT) expression and O-GlcNAcylation in breast Cancer. Mol Cancer Res. (2015) 13:923–33. 10.1158/1541-7786.MCR-14-0536 25636967 PMC4433402

[37] Zhang X Qiao Y Wu Q Chen Y Zou S Liu X. The essential role of YAP O-GlcNAcylation in high-glucose-stimulated liver tumorigenesis. Nat Commun. (2017) 8:15280. 10.1038/ncomms15280 28474680 PMC5424161

[38] Xia H Dai X Yu H Zhou S Fan Z Wei G. EGFR-PI3K-PDK1 pathway regulates YAP signaling in hepatocellular carcinoma: the mechanism and its implications in targeted therapy. Cell Death Dis. (2018) 9:269. 10.1038/s41419-018-0302-x 29449645 PMC5833379

[39] Zhao Y Montminy T Azad T Lightbody E Hao Y SenGupta S. PI3K positively regulates YAP and TAZ in mammary tumorigenesis through multiple signaling pathways. Mol Cancer Res. (2018) 16:1046–58. 10.1158/1541-7786.MCR-17-0593 29545474

[40] Tumaneng K Schlegelmilch K Russell RC Yimlamai D Basnet H Mahadevan N. YAP mediates crosstalk between the Hippo and PI(3)K–TOR pathways by suppressing PTEN via miR-29. Nat Cell Biol. (2012) 14:1322–9. 10.1038/ncb2615 23143395 PMC4019071

[41] Zhang B Zhou P Li X Shi Q Li D Ju X. Bitterness in sugar: O-GlcNAcylation aggravates pre-B acute lymphocytic leukemia through glycolysis via the PI3K/Akt/c-Myc pathway. Am J Cancer Res. (2017) 7:1337–49. 28670495 PMC5489782

[42] Onodera Y Nam J-M Bissell MJ. Increased sugar uptake promotes oncogenesis via EPAC/RAP1 and O-GlcNAc pathways. J Clin Invest. (2014) 124:367–84. 10.1172/JCI63146 24316969 PMC3871217

[43] Diehl JA Cheng M Roussel MF Sherr CJ. Glycogen synthase kinase-3β regulates cyclin D1 proteolysis and subcellular localization. Genes Dev. (1998) 12:3499–511. 9832503 10.1101/gad.12.22.3499 PMC317244

[44] Kanwal S Fardini Y Pagesy P N'tumba-Byn T Pierre-Eugène C Masson E. O-GlcNAcylation-inducing treatments inhibit estrogen receptor α expression and confer resistance to 4-OH-tamoxifen in human breast cancer-derived MCF-7 cells. PLoS ONE (2013) 8:e69150. 10.1371/journal.pone.0069150 23935944 PMC3730543

[45] Krześlak A Józwiak P Lipinska A. Down-regulation of β-N-acetyl-D-glucosaminidase increases Akt1 activity in thyroid anaplastic cancer cells. Oncol Rep. (2011) 26:743–9. 10.3892/or.2011.1333 21637923

[46] Jones DR Keune W-J Anderson KE Stephens LR Hawkins PT Divecha N. The hexosamine biosynthesis pathway and O-GlcNAcylation maintain insulin-stimulated PI3K-PKB phosphorylation and tumour cell growth after short-term glucose deprivation. FEBS J. (2014) 281:3591–608. 10.1111/febs.12879 24938479

[47] Zhang N Chen X. Potential role of O-GlcNAcylation and involvement of PI3K/Akt1 pathway in the expression of oncogenic phenotypes of gastric cancer cells *in vitro*. Biotechnol Appl Biochem. (2016) 63:841–51. 10.1002/bab.1441 26333304

[48] Zhang P Wang C Ma T You S. O-GlcNAcylation enhances the invasion of thyroid anaplastic cancer cells partially by PI3K/Akt1 pathway. Onco Targets Ther. (2015) 8:3305–13. 10.2147/OTT.S82845 26635480 PMC4646590

[49] Manning BD Cantley LC. AKT/PKB signaling: navigating downstream. Cell (2007) 129:1261–74. 10.1016/j.cell.2007.06.009 17604717 PMC2756685

[50] Soesanto YA Luo B Jones D Taylor R Gabrielsen JS Parker. Regulation of Akt signaling by O-GlcNAc in euglycemia. Am J Physiol Endocrinol Metab. (2008) 295:E974–980. 10.1152/ajpendo.90366.2008 18728220 PMC2575895

[51] Phoomak C Silsirivanit A Park D Sawanyawisuth K Vaeteewoottacharn K Wongkham C. O-GlcNAcylation mediates metastasis of cholangiocarcinoma through FOXO3 and MAN1A1. Oncogene (2018). 10.1038/s41388-018-0366-1. [Epub ahead of print]. 29915392 PMC6151127

[52] Ishimura E Nakagawa T Moriwaki K Hirano S Matsumori Y Asahi M. Augmented O-GlcNAcylation of AMP-activated kinase promotes the proliferation of LoVo cells, a colon cancer cell line. Cancer Sci. (2017) 108:2373–82. 10.1111/cas.13412 28973823 PMC5715261

[53] Ferrer CM Lynch TP Sodi VL Falcone JN Schwab LP Peacock DL. O-GlcNAcylation regulates cancer metabolism and survival stress signaling via regulation of the HIF-1 pathway. Mol Cell (2014) 54:820–31. 10.1016/j.molcel.2014.04.026 24857547 PMC4104413

[54] Dienstmann R Rodon J Serra V Tabernero J. Picking the point of inhibition: a comparative review of PI3K/AKT/mTOR pathway inhibitors. Mol Cancer Ther. (2014) 13:1021–31. 10.1158/1535-7163.MCT-13-0639 24748656

[55] Kwei KA Baker JB Pelham RJ. Modulators of sensitivity and resistance to inhibition of PI3K identified in a pharmacogenomic screen of the NCI-60 human tumor cell line collection. PLoS ONE (2012) 7:e46518. 10.1371/journal.pone.0046518 23029544 PMC3460918

[56] Lehman DM Fu D-J Freeman AB Hunt KJ Leach RJ Johnson-Pais T. A single nucleotide polymorphism in MGEA5 encoding O-GlcNAc-selective N-acetyl-beta-D glucosaminidase is associated with type 2 diabetes in Mexican Americans. Diabetes (2005) 54:1214–21. 10.2337/diabetes.54.4.1214 15793264

[57] Zhang H Jia Y Cooper JJ Hale T Zhang Z Elbein SC. Common variants in glutamine:fructose-6-phosphate amidotransferase 2 (GFPT2) gene are associated with type 2 diabetes, diabetic nephropathy, and increased GFPT2 mRNA levels. J Clin Endocrinol Metab. (2004) 89:748–55. 10.1210/jc.2003-031286 14764791

[58] Fricovsky ES Suarez J Ihm S-H Scott BT Suarez-Ramirez JA Banerjee I. Excess protein O-GlcNAcylation and the progression of diabetic cardiomyopathy. Am J Physiol Regul Integr Comp Physiol. (2012) 303:R689–99. 10.1152/ajpregu.00548.2011 22874425 PMC3469670

[59] Springhorn C Matsha TE Erasmus RT Essop MF. Exploring leukocyte O-GlcNAcylation as a novel diagnostic tool for the earlier detection of type 2 diabetes mellitus. J Clin Endocrinol Metab. (2012) 97:4640–9. 10.1210/jc.2012-2229 23066116

[60] Park K Saudek CD Hart GW. Increased expression of β-N-acetylglucosaminidase in erythrocytes from individuals with pre-diabetes and diabetes. Diabetes (2010) 59:1845–50. 10.2337/db09-1086 20413512 PMC2889787

[61] Ruan H-B Han X Li M-D Singh JP Qian K Azarhoush S. O-GlcNAc transferase/host cell factor C1 complex regulates gluconeogenesis by modulating PGC-1α stability. Cell Metab. (2012) 16:226–37. 10.1016/j.cmet.2012.07.006 22883232 PMC3480732

[62] Shi H Munk A Nielsen TS Daughtry MR Larsson L Li S. Skeletal muscle O-GlcNAc transferase is important for muscle energy homeostasis and whole-body insulin sensitivity. Mol Metab. (2018) 11:160–77. 10.1016/j.molmet.2018.02.010 29525407 PMC6001359

[63] Dentin R Hedrick S Xie J Yates J Montminy M. Hepatic glucose sensing via the CREB coactivator CRTC2. Science (2008) 319:1402–5. 10.1126/science.1151363 18323454

[64] Vosseller K Wells L Lane MD Hart GW. Elevated nucleocytoplasmic glycosylation by O-GlcNAc results in insulin resistance associated with defects in Akt activation in 3T3-L1 adipocytes. Proc Natl Acad Sci USA. (2002) 99:5313–8. 10.1073/pnas.072072399 11959983 PMC122766

[65] McClain DA Lubas WA Cooksey RC Hazel M Parker GJ Love DC. Altered glycan-dependent signaling induces insulin resistance and hyperleptinemia. Proc Natl Acad Sci USA. (2002) 99:10695–9. 10.1073/pnas.152346899 12136128 PMC125016

[66] Sano H Kane S Sano E Mîinea CP Asara JM Lane WS. Insulin-stimulated phosphorylation of a Rab GTPase-activating protein regulates GLUT4 translocation. J Biol Chem. (2003) 278:14599–602. 10.1074/jbc.C300063200 12637568

[67] Parker GJ Lund KC Taylor RP McClain DA. Insulin resistance of glycogen synthase mediated by o-linked N-acetylglucosamine. J Biol Chem. (2003) 278:10022–7. 10.1074/jbc.M207787200 12510058

[68] Macauley MS Bubb AK Martinez-Fleites C Davies GJ Vocadlo DJ Elevation of global O-GlcNAc Levels in 3T3-L1 adipocytes by selective inhibition of O-GlcNAcase does not induce insulin resistance. J Biol Chem. (2008) 283:34687–95. 10.1074/jbc.M804525200 18842583 PMC3259902

[69] Macauley MS Shan X Yuzwa SA Gloster TM Vocadlo DJ. Elevation of global O-GlcNAc in rodents using a selective O-GlcNAcase inhibitor does not cause insulin resistance or perturb glucohomeostasis. Chem Biol. (2010) 17:949–58. 10.1016/j.chembiol.2010.07.005 20851344 PMC2954292

[70] Arias EB Kim J Cartee GD. Prolonged incubation in PUGNAc results in increased protein O-Linked glycosylation and insulin resistance in rat skeletal muscle. Diabetes (2004) 53:921–30. 10.2337/diabetes.53.4.921 15047606

[71] Mehdy A Morelle W Rosnoblet C Legrand D Lefebvre T Duvet S. PUGNAc treatment leads to an unusual accumulation of free oligosaccharides in CHO cells. J Biochem. (2012) 151:439–46. 10.1093/jb/mvs012 22337894

[72] Dehennaut V Lefebvre T. Proteomics and PUGNAcity will overcome questioning of insulin resistance induction by nonselective inhibition of O-GlcNAcase. Proteomics (2013) 13:2944–6. 10.1002/pmic.201300363 23983178

[73] Akimoto Y Hart GW Wells L Vosseller K Yamamoto K Munetomo E. Elevation of the post-translational modification of proteins by O-linked N-acetylglucosamine leads to deterioration of the glucose-stimulated insulin secretion in the pancreas of diabetic Goto-Kakizaki rats. Glycobiology (2007) 17:127–40. 10.1093/glycob/cwl067 17095531

[74] Alejandro EU Bozadjieva N Kumusoglu D Abdulhamid S Levine H Haataja L. Disruption of O-linked N-acetylglucosamine signaling induces ER stress and β-cell failure. Cell Rep. (2015) 13:2527–38. 10.1016/j.celrep.2015.11.020 26673325 PMC4839001

[75] Vaidyanathan K Wells L. Multiple tissue-specific roles for the O-GlcNAc post-translational modification in the induction of and complications arising from type II diabetes. J Biol Chem. (2014) 289:34466–71. 10.1074/jbc.R114.591560 25336652 PMC4263854

[76] Ma J Hart GW. Protein O-GlcNAcylation in diabetes and diabetic complications. Expert Rev Proteomics (2013) 10:365–80. 10.1586/14789450.2013.820536 23992419 PMC3985334

[77] Luo B Soesanto Y McClain DA. Protein modification by O-linked GlcNAc reduces angiogenesis by inhibiting Akt activity in endothelial cells. Arterioscler Thromb Vasc Biol. (2008) 28:651–7. 10.1161/ATVBAHA.107.159533 18174452 PMC2734484

[78] Zoccali C Endothelial dysfunction, nitric oxide bioavailability, and asymmetric dimethyl arginine. In: Cardiorenal Syndrome: Mechanisms, Risk and Treatment. Berbari AE, Mancia G, editors. Milano: Springer Milan (2010). p. 235–244. 10.1007/978-88-470-1463-3_17

[79] Ritchie SA Kohlhaas CF Boyd AR Yalla KC Walsh K Connell JMC. Insulin-stimulated phosphorylation of endothelial nitric oxide synthase at serine-615 contributes to nitric oxide synthesis. Biochem J. (2010) 426:85–90. 10.1042/BJ20091580 19925457

[80] Karar J Maity A. PI3K/AKT/mTOR Pathway in Angiogenesis. Front Mol Neurosci. (2011) 4:51 10.3389/fnmol.2011.00051 22144946 PMC3228996

[81] Heath JM Sun Y Yuan K Bradley WE Litovsky S Dell'Italia LJ Activation of AKT by O-GlcNAcylation induces vascular calcification in diabetes. Circ Res. (2014) 114:1094–102. 10.1161/CIRCRESAHA.114.302968 24526702 PMC4030422

[82] Cohen-Solal KA Boregowda RK Lasfar A. RUNX2 and the PI3K/AKT axis reciprocal activation as a driving force for tumor progression. Mol Cancer (2015) 14:137. 10.1186/s12943-015-0404-3 26204939 PMC4513933

[83] Ma X Li H He Y Hao J. The emerging link between O-GlcNAcylation and neurological disorders. Cell Mol Life Sci. (2017) 74:3667–86. 10.1007/s00018-017-2542-9 28534084 PMC11107615

[84] Yuzwa SA Macauley MS Heinonen JE Shan X Dennis RJ He Y. A potent mechanism-inspired O-GlcNAcase inhibitor that blocks phosphorylation of tau *in vivo*. Nat Chem Biol. (2008) 4:483–90. 10.1038/nchembio.96 18587388

[85] Yuzwa SA Shan X Macauley MS Clark T Skorobogatko Y Vosseller K. Increasing O-GlcNAc slows neurodegeneration and stabilizes tau against aggregation. Nat Chem Biol. (2012) 8:393–9. 10.1038/nchembio.797 22366723

[86] Borghgraef P Menuet C Theunis C Louis JV Devijver H Maurin H Increasing brain protein O-GlcNAcylation mitigates breathing defects and mortality of Tau.P301L mice. PLoS ONE (2013) 8:e0084442 10.1371/journal.pone.0084442 PMC3871570 24376810

[87] Yu Y Zhang L Li X Run X Liang Z Li Y. Differential effects of an O-GlcNAcase inhibitor on tau phosphorylation. PLoS ONE (2012) 7:e0035277. 10.1371/journal.pone.0035277 22536363 PMC3334936

[88] Wani WY Ouyang X Benavides GA Redmann M Cofield SS Shacka JJ. O-GlcNAc regulation of autophagy and α-synuclein homeostasis; implications for Parkinson's disease. Mol Brain (2017) 10:32. 10.1186/s13041-017-0311-1 28724388 PMC5517830

[89] Marotta NP Lin YH Lewis YE Ambroso MR Zaro BW Roth MT. O-GlcNAc modification blocks the aggregation and toxicity of the Parkinson's disease associated protein α-synuclein. Nat Chem. (2015) 7:913–20. 10.1038/nchem.2361 26492012 PMC4618406

[90] Zhang J Lei H Chen Y Ma Y-T Jiang F Tan J. Enzymatic O-GlcNAcylation of α-synuclein reduces aggregation and increases SDS-resistant soluble oligomers. Neurosci Lett. (2017) 655:90–4. 10.1016/j.neulet.2017.06.034 28673834

[91] Gorman AM. Neuronal cell death in neurodegenerative diseases: recurring themes around protein handling. J Cell Mol Med. (2008) 12:2263–80. 10.1111/j.1582-4934.2008.00402.x 18624755 PMC4514105

[92] Parween S Varghese DS Ardah MT Prabakaran AD Mensah-Brown E Emerald BS. Higher O-GlcNAc levels are associated with defects in progenitor proliferation and premature neuronal differentiation during *in-vitro* human embryonic cortical neurogenesis. Front Cell Neurosci. (2017) 11:e00415. 10.3389/fncel.2017.00415 29311838 PMC5742625

